# Optimizing Biodegradable Starch-Based Composite Films Formulation for Wound-Dressing Applications

**DOI:** 10.3390/mi13122146

**Published:** 2022-12-04

**Authors:** Mohammad Mohsen Delavari, Ixchel Ocampo, Ion Stiharu

**Affiliations:** 1 Department of Mechanical, Industrial, and Aerospace Engineering, Concordia University, Montreal, QC H3G 1M8, Canada; 2 School of Engineering and Sciences, Tecnológico de Monterrey, Av. Eugenio Garza Sada 2501 Sur, Monterrey 64849, Mexico

**Keywords:** wound dressings, machine learning, starch, PVA, biodegradable, optimization

## Abstract

This paper utilizes response surface methodology (RSM) design-based analyses to optimize starch-based wound dressings that were characterized based on weight loss (WL%), swelling index (SI%), and mechanical strength (TS). The wound-dressing materials were prepared by employing a modified casting method, using various concentrations of starch (0.5–2 *w*/*w*%), polyvinyl alcohol (PVA) (0.5–2 weight%), citric acid (1.5–4 weight%), and glycerol (1.5–4 weight%) to yield wound-dressing films with appropriate combinations of in vitro degradation, swelling index, and tensile strength. As a result of the response surface method-based analysis, the swelling index, in vitro degradation, and tensile strength were linearly related to variations in the formulation of organic components. Based on our experimental investigations, the optimized film (formulation: 1 weight% PVA, 2 weight% starch, 1.5 weight% citric acid, and 1.5 weight% glycerol) exhibited an outstanding swelling index (343.52%), suitable in vitro degradation (53.22%), and excellent tensile strength (8.82 MPa). The response surface plots for the dependent variables, swelling index (SI%), weight loss (WL%), and mechanical strength (TS), showed that in all dual relations, the PVA-starch combination significantly affected all dependent variables; however, the PVA-citric acid interaction showed the most excellent effect on the swelling index. As a result, every component of the resulting film had a lesser amount of all ingredients to achieve better properties at a lower material cost. Starch-based/PVA films have been identified in this paper as optimal and more affordable wound-dressing films.

## 1. Introduction

The consumption of synthetic polymers is linked with environmental problems; therefore, renewable, and eco-friendly natural polymers have started to be used to try to avoid the high costs and hazards associated with the recycling and disposal of plastics [1,2,3]. Despite the fact that these renewable polymers are biodegradable, they are extremely expensive to integrate into existing economies [4,5].

A transitional phase is inevitable in creating composites from both natural and synthetic polymers. Making use of such combinations provides viable solutions for enhancing the biodegradability of synthetic polymers. Conventional and frontier research in the field of biocomposite films for biomedical applications focuses on developing inexpensive, easily fabricated, highly biodegradable blends with satisfactory mixtures of characterization parameters [6].

Because starch is easily produced from sustainable natural biological resources, such as corn, potato, wheat, and rice, it is among the most affordable and feasible natural polymers. This is as compared to chitosan, collagen, gelatin, and alginate. In terms of synthetic polymer-based materials and biocomposites, starch is the most viable and cost-effective textile available [2,3,6,7].

Nevertheless, starch suffers from fundamental limitations regarding its processability and shape stability in fluids due to its high solubility in water and insignificant physicomechanical characteristics. Because native starch is challenging to work with directly, it must be blended with appropriate synthetic materials to create the desired results [8,9]. Gelatinization, crosslinked phosphorylation, and hydroxy-propylation are commonly used techniques in this regard [6]. In order to enhance the properties of polymer composite films for commercial purposes, these methods facilitate the fusion and crosslinking of starch [2,3,8,9,10].

As opposed to polylactic-co-glycolic acid, polycaprolactone, and polyglycolide, polyvinyl alcohol (PVA) is the most economical synthetic biodegradable polymer for use in biomedical applications [11,12]. The cost of polyethylene glycol is lower than the cost of these expensive synthetic polymers; however, it is not biodegradable [13]. It has also been found that PVA provides an improved degradation of biocomposites. Furthermore, PVA is an FDA-approved biomaterial, and its isotactic material is predisposed to degradation due to its stereo-chemical composition [3,14].

Hydrophilic, non-toxic, and easy-to-process, starch-based/PVA composite biomaterials are commonly used as scaffolding support materials for tissue engineering. Overall, the starch-based/PVA composite films exhibit superior biodegradability, mechanical and pH stability, flexibility, and semi-permeability, compared to all other synthetic polymer-derived materials [2,3,10,15,16], enabling oxygen and nutrients to be transported to the wound bed area for the critical survival of biological cells [17].

As a result of inflammation, proliferation, and remodeling, wound healing is a complex process characterized by three classic phases. The length of the wound can vary, depending on its severity and type [18]. Acute wounds generally heal within 8–12 weeks, following all the phases mentioned above [19]. Deficient signals result in chronic wounds, extending the inflammatory process. Consequently, chronic wounds pose a major concern for clinicians since they typically take a long time to heal [18,20].

Over the last few decades, it has become increasingly apparent that a moist environment promotes wound healing due to the stimulation of keratinocyte migration, resulting in more rapid wound healing [21]. A wound dressing should not only be biocompatible and protect the wound but also maintain wound hydration, remove excessive exudate, allow oxygen exchange with the environment, and physically protect against infections. The material should also be flexible and possess high tensile strength, as well as specific mechanical properties [22].

Various wound dressings are available, including films, hydrogels, hydro-colloids, hydro-actives, foams, alginates, and hydro-fibers [18,23]. One type may be more appropriate for a particular wound based on its depth and the amount of exudate [18]. Using films for direct drug delivery to wound locales is possible because they are gas-permeable but liquid and bacteria-impervious. Previous research shows customized polymeric blends are targeted for preparing new wound-dressing materials, emphasizing the polymer’s specific function. By changing the composition of fibrin/chitosan composite sheets within the hydrogel films, Devi et al. manufactured fibrin/chitosan composite sheets. As chitosan decreased the tensile strength of hydrogel films, sodium alginate was added in various proportions. This enhances the mechanical properties of these films [24,25].

Based on the available applied research in a wide variety of starch/PVA composite films containing nanoparticles and natural additives, it is evident that additives play an integral role in these films. As an additive to such hydrogels, alternative crosslinkers or plasticizers are added to reduce mechanical instability [8]. With the addition of zinc oxide nanoparticles (nZnO), Baghaie et al. improved PVA/starch/chitosan hydrogels’ healing characteristics and tensile strength [26]. Similarly, Delavari et al. embedded nZnO particles into starch-based material, resulting in a novel biodegradable and antibacterial starch-based wound dressing, improving its physical and in vitro degradation characteristics [15,16]. To achieve the controlled release of erythromycin, Tavakoli et al. prepared a PVA/honey hydrogel using a borax crosslinker [27]. Using PVA hydrogel membranes with different concentrations of hyaluronic acid and aloe vera, Fahmy et al. and Hajian et al. examined their degradation properties [28,29]. It was observed that weight loss for both composites was steady for up to 27 days. Ahmed et al. also reported a stable increase in drug release for 24 h for the samples [30].

By varying the amounts of the additives (citric acid (CA) and/or glycerol (Gl), Yoon et al. [31] targeted the swelling degree, tensile strength, elongation, and solubility. According to Wu et al. [32], the composite films exhibited enhanced antibacterial activity when CA was included in the mixture, along with all other constituents (PVA, starch, CA, and Gl). Previously, our group investigated the effects of CA on the swelling index, solubility, gel fraction (GF), tensile strength, elongation, WVTR, and antibacterial effectiveness of starch-based wound-dressing films [3,33]. Consequently, CA is a supposed acid with properties such as crosslinking [2,10,34], plasticizing [8], and antibacterial effects [32]. In addition, it can also be utilized as an additive to further optimize and enhance the wound-dressing film characteristics of starch-based wound dressings at a low cost.

It has been well established that a critical component or additive in a starch-based composite matrix can significantly affect the characterization parameters. It has been challenging to target the compositional optimality and synergistic effects when considering systematic variations in the compositions of all constituents. According to our recent research, starch-based materials behave similarly in most cases when increments of citric acid are added, but this is not always the case [3]. Ahmed et al. reported that the composite has an optimal gel fraction and tensile strength, but they did not consider whether the two composites are similar or different [30].

There was a lack of mathematical models and formulations for analyzing the complexities related to the relationships between various characterization parameters, such as tensile strength, weight loss percentage, and swelling index. Intending to fill the above-summarized gaps in the literature, this paper examines the optimal combination of highly complex ingredients (PVA, starch, citric acid, and glycerol) during the synthesizing of starch-based/PVA wound dressings to derive the correlation between such components of the starch-based composites and the essential characteristics of a wound-dressing material and to identify the most suitable formulation of wound-dressing materials.

## 2. Materials and Methods

### 2.1. Materials

Sigma Aldrich, Canada, provided the polyvinyl alcohol (M_w_ 20,000–23,000 g/mol and 88% hydrolyzed), potato starch (M_w_ 342.30 g/mol), and citric acid (M_w_ 192.12 g/mol). The glycerol (M = 92.05 g/mol; purity = 99.0%) was purchased from Fisher Scientific. The deionized water (DI) was collected from a standard setup. DI water equipment provided deionized water (DI) in our laboratory (MIAE Department) at Concordia University.

### 2.2. Preparation of the Films

Our previous article explained how the starch-based/PVA blended solution used in this study is prepared [3]. An experimental design, based on a statistical technique, was used to determine the concentration variation of the formulation in the composite solution (0.5 ≤ PVA ≤ 2.5, 0.5 ≤ starch ≤ 2.5, 0.25 ≤ CA ≤ 5.25, and 0.25 ≤ glycerol ≤ 5.25). Furthermore, the solution was poured onto a glass plate. Our research group dried the cast composite films using the optimized process described in the previous article [3]. Based on the insights offered in the relevant literature [2,3,8,9,10], we chose the concentration range of the different independent variables. In this study, 30 sets of films were prepared with various combinations of constituents (Table 1) and then characterized in terms of SI, weight loss, and tensile strength (all samples were analyzed five times, and the standard deviation value was ≤ ±5).

### 2.3. Design and Analysis of the Experiments

As part of the statistical design of experiments, a response surface methodology described by central composite design (CCD) has been used. For the independent variables, starch concentration (St), PVA concentration, citric acid concentration (CA), and glycerol concentration (Gl) were considered, and for the dependent variables, the swelling index (SI), in vitro degradation (WL), and mechanical strength (TS) were evaluated. Dressing materials must have a maximum SI value to prevent wound fluid accumulation, which can worsen contamination. In order to avoid infection and enable localized antibacterial effects, the degradation rate represents the amount of weight lost by the composite film matrix due to component degradation.

As wound dressing materials need moderate mechanical strength in order to be easily handled and applied to wound surfaces, they need to have a minimum degree of shape stability in fluids. In order to fabricate PVA composite films that address these issues, they were considered the most relevant and appropriate dependent variables. Several sources provided insight and a learning process, determining the independent variable ranges. In this study, each dependent variable was analyzed using multiple regression. Minitab 19.0 provided the coefficients of significant terms by using an ANOVA (Analysis of Variance) in which the regressed data’s F-values confirmed the terms’ significance.

The accuracy of assessing model fitness was also assessed by evaluating R^2^.

### 2.4. Swelling Index

For the purposes of evaluating the fluid absorption capacity of these films, it is necessary to apply the swelling index methodology. As part of this method, samples (1 × 1 cm^2^) were immersed in phosphate buffer saline (PBS) solution and incubated at 37 °C for 24 h. The swelling index (SI%) is calculated utilizing Equation (1); the test procedure followed that in our previous articles [3,16].

(1) $$\mathrm{SI}=\frac{W_{s}}{W_{0}}\times 100\%$$

W_0_ and Ws are the dry and wet weights after immersion in a PBS solution.

### 2.5. Weight Loss

We evaluated the weight loss after 14 days of immersion in PBS solution with 7.4 pH at 37 °C, using in vitro degradation methods. The weight-loss percentage value can be determined using Equation (2). Our previous articles outlined the procedure for conducting the test [3,16]:

(2) $$\mathrm{WL}\%=\frac{W_{0}-{\;W}_{f}}{W_{0}}\times 100\%$$

where W_0_ is the dry weight of the samples prior to immersion in the PBS media or saline, and W_f_ is the weight of the samples following drying at 37 °C.

### 2.6. Mechanical Strength

In order to ensure that the prepared films are durable and flexible enough to be used as wound dressings and wearable electronics, we developed a high-resolution testing device [15]. The ASTM D882–10 test was conducted following the procedure in our previous article [16].

## 3. Results

### 3.1. Swelling Index

For the given constraints, a first-order equation best represented the resulting mean data values of the SI percentage. It is appropriate to use the following expression:

SI (%) = 605.6 − 45.8 PVA − 53.3 Starch − 34.35 CA − 42.48 Gl(3)

where the swelling index, called SI, is determined by PVA, starch, citric acid (CA), and glycerol concentration, respectively.

A positive parametric value indicates that the SI percentage will increase with the dependent variable, whereas a negative value indicates the opposite. The SI activity reached the maximum value, with starch at 1 wt %, PVA at 1 wt %, CA at 1.5 wt %, and Gl at 1.5 wt %. An analysis of the ANOVA results of the above model indicated its promising features, which included a low *p*-value (<0.0001) and a high F-value (19.55), implying that the model is significant (Table 2). The regression equation yielded a determination coefficient of 0.7190 (R^2^), suggesting that the statistical model can explain 71.90% and 62.78% of the experimental and prediction data variability in the responses, respectively.

### 3.2. In Vitro Degradation

As a result of the given constraints, a first-order equation was found to be best suited for representing the mean values of the WL percentage. The relevant expression is as follows:

WL = 30.82 − 12.66 PVA + 11.82 Starch + 5.885 CA + 1.403 Gl (wt %)(4)

where weight loss, called WL, is determined by PVA, starch, citric acid (CA), and glycerol concentration, respectively.

The WL percentages had the highest values, with starch at 1 wt %, PVA at 2 wt %, CA at 4 wt %, and Gl at 4 wt %. As can be seen from Table 3, the model fitness parameters indicate a high degree of fitness (*p*-value < 0.0001) and a high F-value (44.03). This suggests that the model fitness degree is highly significant. As a result of the ANOVA, the regression equation yielded a determination coefficient of 0.8558 (R^2^) and an 82.72% predicted R^2^ value.

### 3.3. Mechanical Strength

A first-order equation was found to be the most appropriate method by which to represent the resulting mean data values of tensile strength values according to the particular requirements. As a result, the following expression is appropriate:

TS = 12.01 + 0.723 PVA − 1.732 Starch − 1.827 CA − 0.157 Gl(5)

where tensile strength, called TS, is determined by PVA, starch, citric acid (CA), and glycerol concentration (Gl), respectively.

The mechanical strength reached the highest value at starch 2 wt %, PVA 1 wt %, CA 1.5 wt %, and Gl 1.5 wt %. As can be seen from Table 4, the model fitness parameters indicate a high degree of fitness (*p*-value < 0.0001) and a high F-value (34.97). This suggests that the model fitness degree is highly significant. As a result of ANOVA, the regression equation yielded a determination coefficient of 0.8484 (R^2^), suggesting that the statistical model can explain 84.84% and 77.30% of the experimental and prediction data variability in the responses, respectively.

### 3.4. Response Surface Graphs

**Figure 1:**
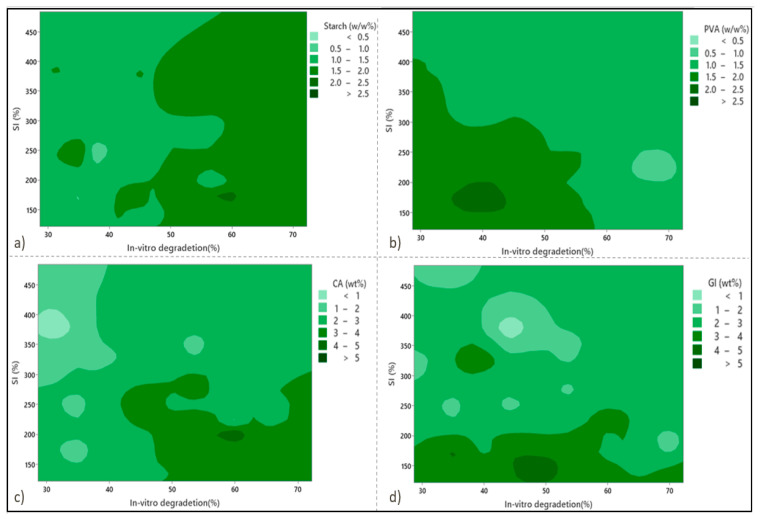
Contour plots show the effects of the independent variables on the swelling index (SI%) and weight loss (WL%) in (**a**) starch, (**b**) PVA, (**c**) citric acid, and (**d**) glycerol.

In order to design the statistical experiments, a response surface methodology was applied. Figure 1 shows the contour plots for SI against the WL at zero level against independent variables PVA concentration, starch concentration (St), citric acid concentration (CA), and glycerol concentration (Gl). As seen in Figure 1a,c, by increasing the starch and citric acid content values in the preparation formula, the WL percentages increase significantly. Moreover, Figure 1a shows that the WL and SI values increase remarkably by increasing the starch content. Experimental investigations have determined the maximum and minimum values of SI% to be 483.25% and 121.58%, respectively. Based on the available literature guidelines, these values are consistent with a swelling index value greater than 260%, indicating the presence of superabsorbent wound-dressing films [16,30].

As shown in Figure 1b, at the higher values of PVA (> 10 wt %), the minimum values for both SI and WL values were achieved, especially in the case of the WL values. The reason for this is due to an increase in polymer intensity in the substance structure, which promotes crosslinking and reduces porosity. A marginal drop in SI% can be observed for either the highest PVA content or the highest starch content point. Regarding the effects of glycerol content on the SI percentage, it is demonstrated in Figure 1d that glycerol enables a drastic decline in SI%. Three hydroxyl groups (OH) in glycerol facilitate hydrogen bonding with the hydroxyl groups of PVA and starch, leading to higher crosslinking capabilities and a lower SI%. The effects of glycerol and citric acid tend to promote WL percentage values, with citric acid having a more significant impact than glycerol (Figure 1c,d).

**Figure 2:**
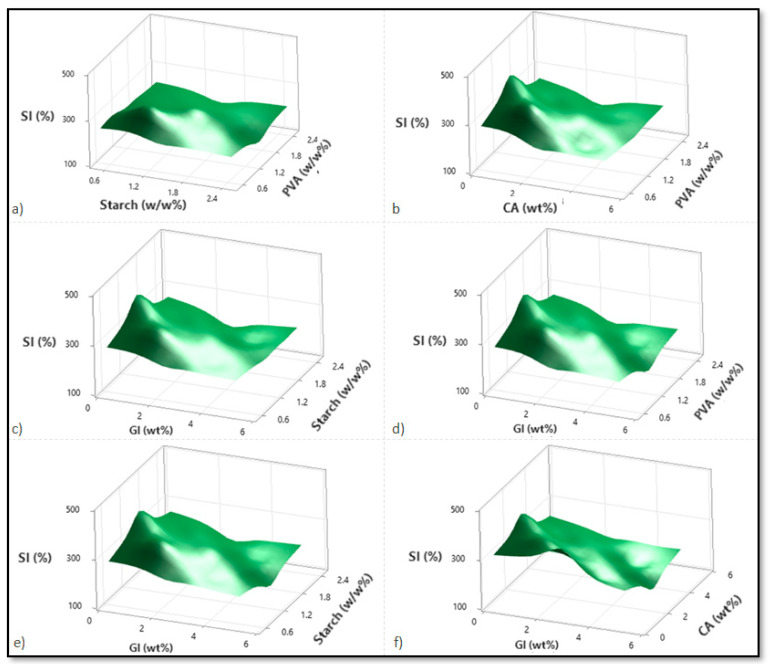
Corresponding response surface plots show the independent variables’ effects on the swelling index (%). (**a**) Starch-PVA, (**b**) Citric acid-PVA, (**c**) Glycerol-Starch, (**d**) Glycerol-PVA, (**e**) Glycerol-Starch, and (**f**) Glycerol-Citric acid.

**Figure 3:**
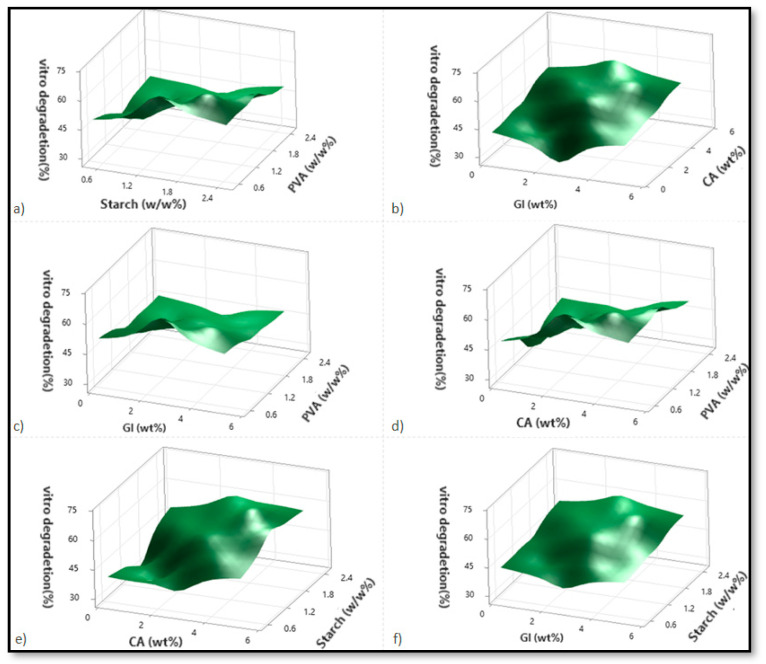
The corresponding response surface plots show the independent variables’ effects on weight loss (WL%). (**a**) Starch-PVA, (**b**) Glycerol-Citric acid, (**c**) Glycerol-PVA, (**d**) Citric acid-PVA, (**e**) Citric acid-Starch, and (**f**) Glycerol-Starch.

**Figure 4:**
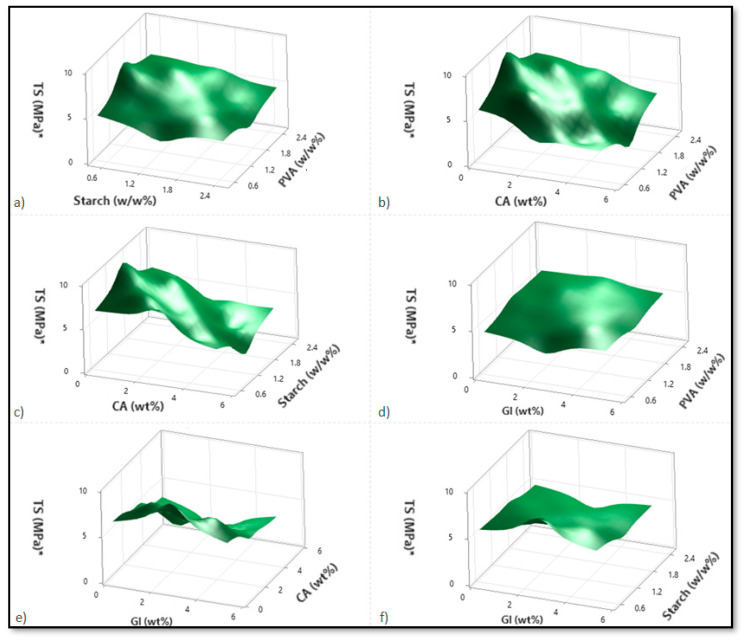
Corresponding response surface plots show the independent variables’ effects on tensile strength (TS (MPa)). (**a**) Starch-PVA, (**b**) Citric acid-PVA, (**c**) Citric acid-Starch, (**d**) Glycerol-PVA, (**e**) Glycerol-Citric acid, and (**f**) Glycerol-Starch.

Based on variations in the PVA, starch, CA, and Gl concentrations, Figure 2, Figure 3 and Figure 4 illustrate the corresponding response surface plots for the dependent variables, such as swelling index (SI%), weight loss (WL%) or in vitro degradation, and mechanical strength (TS).

According to Figure 2a–f, all four components reduced the SI%; only CA and PVA reduced the SI% significantly. Among the dual interactions, the interactions between PVA over starch and glycerol over citric acid were predominant. As indicated by the individual variable terms, all four independent variables demonstrated a similarly negative effect. For the PVA–starch scenario, both polymers significantly reduced the SI%. This is due to increased polymer values within the material structure, which enhance crosslinking and decrease permeability (Figure 2a). At higher CA intensities, increasing the PVA concentration did not affect SI%; however, at lower CA dilutions, increasing PVA concentration initially increased and then started reducing the SI%.

Additionally, citric acid content enhancement reduced SI% in terms of lower and higher PVA concentrations, with a more significant reduction in the case of higher PVA concentrations (Figure 2b). Despite this finding, at low and high CA concentrations, SI% decreases as starch participates more readily in the esterification reaction than PVA. This can be attributed to the crosslinking influence of CA with PVA or starch (Figure 2c). Glycerol facilitates a considerable drop in SI% for both the PVA–glycerol, and starch–glycerol interactions (Figure 2d,e). The citric acid–glycerol interactions also show a drastic decrease in SI% according to glycerol concentration, confirming glycerol’s predominant influence over citric acid.

In accordance with the literature, the swelling index maintains its constant course after a particular concentration of citric acid increases [16]. Citric acid contains three carboxyl (COOH) groups as a crosslinking agent. Conversely, glycerol containing three hydroxyl groups (OH) facilitates hydrogen bonding with PVA and the starch hydroxyl groups [8]. A comparison between the citric acid carboxyl groups and the glycerol hydroxyl groups shows that the citric acid carboxyl groups form much more stable hydrogen bonds. Citric acid, however, increases with increasing concentration in the polymer matrix, and the free carboxyl groups bond strongly with water as the free citric acid content increases. Due to its strong affinity for water, citric acid will have the advantage in the presence of excess carboxyl and hydroxyl groups. This is significantly different from glycerol, which has only three hydroxyl groups. Thus, this compound is likely to act as a plasticizer because it has a reduced crosslinking ability [8].

Corresponding to Figure 3a–f, only PVA significantly reduced the weight-loss percentage among all four components. A significant increase in the in vitro degradation percentage was observed when the concentration of citric acid and starch was increased but not when glycerol enhancement was used. PVA, however, had a significant negative impact on degradation. Among the dual interactions, citric acid–starch interaction demonstrated the most prominent effect. Due to the increasing solid entity in the substance structure, the weight loss percentage decreased with rising PVA intensity for the starch–PVA sample. As starch is a biodegradable natural polymer, its improvement favors weight loss. As a result, the increase in weight loss percentage was much higher in cases where the starch concentrations were higher than in those where the PVA concentrations were lower (Figure 3a). In the case of dual interaction between citric acid and glycerol, both citric acid and glycerol contribute to enhancing degradation, with citric acid performing more effectively than glycerol. At higher citric acid concentrations, glycerol initially enhances degradation but is subsequently reduced due to the effect of the citric acid on glycerol (Figure 3b).

The weight loss percentage will increase for any PVA intensities when there is an interaction between PVA and citric acid. A decrease in PVA concentration caused more significant degradation due to the lower concentration of PVA. Furthermore, higher PVA concentrations, when combined with lower glycerol or citric acid concentrations, resulted in lower weight loss percentages for citric acid but not for glycerol. This is due to the stronger hydrogen bonds formed by the carboxyl groups of citric acid intermolecular interactions being improved compared to glycerol [8]. Because citric acid forms stronger hydrogen bonds than glycerol, degradation enhancement is more evident at reduced PVA concentrations when the citric acid is increased incrementally. As a result, the residual free citric acid content increases as the concentrations of citric acid increase (Figure 3c,d). This can be explained by the increased content of carboxyl groups associated with water, which is responsible for its increased solubility and plasticizing properties [8].

It was found that the weight-loss percentage increased with the increases in starch and citric acid concentrations and starch-glycerol concentrations, respectively, as well as starch–citric acid interactions. A significant increase in weight loss percentage is seen only for citric acid in the case of starch–citric acid interaction. In the case of a starch–glycerol interaction case, the value increment is higher when the citric acid and starch concentrations are highest. However, in the case of a starch increment, the weight loss percentage profile is higher than in the case of a glycerol increment (Figure 3e,f). According to the regression equation, starch > citric acid > glycerol is the primary contributing factor to weight loss percentage, which is in agreement with the hypothesis. 

It is nevertheless evident from the analysis and a comparison of the PVA–citric acid and starch–citric acid interaction cases that the weight loss percentage value obtained for lower PVA and citric acid concentrations is greater than that obtained for lower starch concentrations and any modification in citric acid content. As a result, citric acid reacts more readily with starch than with PVA, as can be concluded. With a better network structure, the cross-linked starch material reduces degradation. In addition to the higher weight loss percentage value obtained from a lower PVA, the corresponding value obtained with higher starch and citric acid concentrations is also higher than that obtained from lower PVA and high citric acid concentrations. As a result of the lower PVA content, more free citric acid remains, resulting in better degradation (Figure 3a–f).

Figure 4a–f shows the response surface plots for the tensile strength with respect to alterations in two independent variables. On the basis of the response surface plots, tensile strength increased with the PVA; however, it decreased with increases in starch, citric acid, glycerol, and especially citric acid.

Regarding the PVA–starch interactions shown in Figure 4a, enhancing the PVA increases the tensile strength for lower starch concentrations. At high starch concentrations, however, the tensile strength decreases only marginally with increasing PVA. As the concentration of starch increases in higher PVA cases, the tensile strength decreases rapidly. Since starch and PVA do not mix well, adding starch to PVA degrades the blend’s physical properties (tensile strength). As a result, the maximum tensile strength can be achieved at the lowest and highest PVA contents. Likewise, the same reasoning applies to those cases with a higher starch level and a lower PVA level.

The tensile strength decreases with increasing citric acid content for the PVA–citric acid interactions and for both lower and higher PVA contents. Conversely, the tensile strength of the composite increases marginally with an increase in PVA content at both low and high citric acid concentrations (Figure 4b). For the starch–citric acid interaction, tensile strength decreases with increasing starch concentration, regardless of the concentration of citric acid (Figure 4c). In this case, the results obtained for citric acid were similar to those obtained for PVA–citric acid interaction. CA was found to affect both plasticization and crosslinking within the polymer matrix. The CA residue increased in the blends after CA was added to the film solution, which reduced the macromolecule interactions and decreased the tensile strength. As a result of the crosslinking between PVA and starch molecules caused by citric acid, -C=C- groups are formed by the loss of the -OH chains [3,8].

It was found that PVA enhancement increased the tensile strength values in the case of PVA-glycerol interaction and for smaller glycerol concentrations (Figure 4d). In contrast, when glycerol concentrations are high, the tensile strength variation is negligible. In a similar fashion, high PVA concentrations result in a reduction in tensile strength due to the glycerol increments. The influence of glycerol is, however, negligible in the case of low PVA contents. A comparison of PVA–citric acid interactions and PVA–glycerol interactions showed that lower concentrations of PVA and citric acid enhance tensile strength more effectively than lower concentrations of PVA and glycerol.

Additionally, when citric acid and glycerol interact, the effects of glycerol are almost negligible, and primarily citric acid concentration reduces the strength of the tensile bond (Figure 4e). In the case of starch–glycerol interaction, glycerol has a minimal impact. In several instances, starch concentrations also reduced the tensile strength (Figure 4f).

## 4. Discussion

As shown in this paper, by using the modified casting method, we were able to develop starch-based wound dressings that were characterized and optimized based on the swelling index, weight loss, and mechanical strength. As a result of the literature data available, this study has provided helpful insight into the possibility of extending predecessor formulations. According to the optimal data, 1 wt % of PVA, 2 wt % of starch, 1.5 wt % of citric acid, and 1.5 wt % of glycerol is the most effective starch-based film to achieve the desired product characteristics. Compared to the examples in the published literature, the optimal film achieved a very high swelling index of 343.52 ± 4.92%, an acceptable degradation of 53.22 ± 3.21%, and an adequate tensile strength of 8.82 MPa. As a result of the response surface method-based analysis, weight loss, swelling index, and tensile strength were linearly related to variations in the formulation of organic components. However, Das et al. [33] reported optimal film composition with a combination of ingredients at higher levels, with 5 *w*/*w*% of PVA, 10 *w*/*w*% of starch, 15 wt % of citric acid, and 15 wt % of glycerol, along with a longer drying duration (12 h) compared to this work. In their work, among all data sets, a 338.37% swelling index, 53.27% weight loss, and 4.56 MPa tensile strength were obtained, all of which are lower than this work’s results, except for weight loss. Wu et al. [32] also reported higher film ingredient values, at 7.39 *w*/*w*% PVA, 7.39 *w*/*w*% starch, 5.5 wt % citric acid, and 2.62 wt % glycerol; however, they obtained significantly higher tensile strength data (45.54 MPa) and swelling index (5431%) despite the fact that similar experimental procedures were used. As a result, every component of the resulting film had a lesser amount of all ingredients except for PVA to achieve better properties at a lower cost. In this paper, better and more affordable starch/PVA/citric acid/glycerol wound-dressing films have been identified as optimal films.

## 5. Conclusions

As part of the production of starch-based/PVA wound dressings, this paper investigates the optimal combination of the highly complex quadratic ingredients (PVA, Starch, CA, and Gl). In all dual interactions, the PVA–starch mixture significantly influenced all dependent variables; however, the PVA–the citric acid mixture was the most effective for the swelling index scenario. PVA–glycerol and citric acid–glycerol are the second-most influential mixtures on degradation and tensile strength, respectively. Compared to the literature reporting on wound-dressing formulation results, the obtained optimal formulation is more cost-effective. Moreover, for every independent variable, every binary interaction associated with glycerol, with the exception of the swelling index, had a minimal impact and F value. The effect of glycerol on all other measured characteristics was minimal in all responses. In order to identify the most effective PVA-starch composite film composition for wound dressing applications, further understanding of WVTR, in vivo wound healing, cytotoxicity, and cell assay characteristics is also needed.

## Figures and Tables

**Table 1:** Experimental data for central composite design.

n.	PVA (*w*/*w*%)	Starch (*w*/*w*%)	CA (wt %)	Gl (wt %)	SI (%)	In Vitro Degradation (%)	TS (MPa)
1	1.5	1.5	2.5	2.75	251.48 ± 5.75	53.06 ± 1.39	5.18
2	1.5	1.5	2.75	2.75	248.74 ± 3.91	49.95 ± 2.92	4.98
3	2	1	1.5	4	172.52 ± 4.63	35.06 ± 1.06	8.68
4	1.5	1.5	2.75	0.25	375.11 ± 4.25	44.32 ± 2.19	5.69
5	1.5	1.5	0.25	2.75	381.45 ± 5.46	31.22 ± 0.48	9.56
6	1	1	1.5	4	328.94 ± 4.43	38.66 ± 3.9	6.78
7	1.5	1.5	2.75	2.75	283.66 ± 3.89	53.25 ± 4.43	5.69
8	1	2	4	4	121.58 ± 4.32	72.23 ± 2.79	3.12
9	1	2	1.5	1.5	343.52 ± 4.92	53.22 ± 3.21	8.82
10	1.5	2.5	2.75	2.75	171.12 ± 4.34	58.15 ± 1.31	3.25
11	2	2	1.5	1.5	243.25 ± 5.52	35.26 ± 2.07	7.32
12	2	2	4	1.5	236.56 ± 4.63	52.36 ± 1.3	1.69
13	2	2	1.5	4	162.32 ± 4.51	44.25 ± 4.43	4.98
14	2	1	1.5	1.5	319.65 ± 5.23	28.65 ± 1.03	9.68
15	1	1	1.5	1.5	483.25 ± 4.32	33.47 ± 2.68	6.54
16	0.5	1.5	2.75	2.75	228.65 ± 4.95	66.85 ± 4.79	3.43
17	1	2	1.5	4	219.08 ± 4.63	59.98 ± 4.99	8.09
18	2	1	4	1.5	249.56 ± 4.21	45.36 ± 4.9	4.87
19	2.5	1.5	2.75	2.75	179.22 ± 4.89	39.89 ± 4.92	5.76
20	1.5	1.5	5.25	2.75	205.43 ± 4.01	59.96 ± 0.93	1.02
21	1.5	1.5	2.75	5.25	148.56 ± 4.96	48.55 ± 1.34	4.21
22	1.5	0.5	2.75	2.75	241.32 ± 4.89	38.15 ± 1.86	8.12
23	1.5	1.5	2.75	2.75	261.22 ± 3.92	56.39 ± 0.87	4.52
24	1	2	4	1.5	189.52 ± 4.79	69.06 ± 1.34	1.23
25	1	1	4	4	194.26 ± 4.95	56.57 ± 3.76	2.69
26	1.5	1.5	2.75	2.75	261.23 ± 4.92	55.95 ± 2.33	4.68
27	1	1	4	1.5	278.65 ± 4.95	53.89 ± 6.54	2.74
28	2	1	4	4	156.02 ± 4.78	45.5 ± 1.18	4.43
29	1.5	1.5	2.75	2.75	266.32 ± 4.95	56.38 ± 2.8	3.41
30	2	2	4	4	167.89 ± 4.92	52.65 ± 4.94	1.25

**Table 2:** Swelling index output data, with a summary of the ANOVA.

Source	DF *	Sum of Squares	Mean Square	F-Value	*p*-Value
Regression	4	141,566	35,391.6	19.55	0.000
PVA	1	12,599	12,599.1	6.96	0.014
Starch	1	17,042	17,041.6	9.41	0.005
Citric Acid	1	44,250	44,249.7	24.44	0.000
Glycerol	1	67,676	67,676.0	37.38	0.000
Error	25	45,256	1810.3		
Lack-of-Fit	20	44,481	2224.1	14.34	0.004
Pure Error	5	775	155.1		
Total	29	186,823			

* (Degree of Freedom)

**Table 3:** Weight loss output data, with a summary of the ANOVA.

Source	DF *	Sum of Squares	Mean Square	F-Value	*p*-Value
Regression	4	3172.48	793.12	44.03	0.000
PVA	1	961.53	961.53	53.38	0.000
Starch	1	838.39	838.39	46.55	0.000
Citric Acid	1	1298.75	1298.75	72.10	0.000
Glycerol	1	73.82	73.82	4.10	0.054
Error	25	450.30	18.01		
Lack-of-Fit	20	417.43	20.87	3.18	0.102
Pure Error	5	32.87	6.57		
Total	29	3622.78			

* (Degree of Freedom)

**Table 4:** Tensile strength output data, with a summary of the ANOVA.

Source	DF *	Sum of Squares	Mean Square	F-Value	*p*-Value
Regression	4	4	147.270	36.817	34.97
PVA	1	1	3.139	3.139	2.98
Starch	1	1	17.992	17.992	17.09
Citric Acid	1	1	125.218	125.218	118.93
Glycerol	1	1	0.920	0.920	0.87
Error	25	25	26.322	1.053	
Lack-of-Fit	20	20	23.348	1.167	1.96
Pure Error	5	5	2.975	0.595	
Total	29	29	173.592		

* (Degree of Freedom)

## Data Availability

All the results related to the results published in this paper are presented within the article.
